# Self-reference promotes vocabulary learning in a foreign language

**DOI:** 10.3758/s13423-025-02674-w

**Published:** 2025-03-19

**Authors:** Shimon Pruss, Avi Karni, Anat Prior

**Affiliations:** 1 Department of Learning Disabilities, Faculty of Education, University of Haifa, 199 Abba Hushi Blvd., Haifa, Israel; 2 Edmond J. Safra Brain Research Center for the Study of Learning Disabilities, University of Haifa, Haifa, Israel

**Keywords:** Vocabulary, Memory, Encoding, Self, Self-reference effect, L2

## Abstract

Encoding information in reference to the self leads to improved memory, a phenomenon termed the self-reference effect. Learning vocabulary in a foreign language (L2) is a challenging memory task, because learning thousands of words is necessary to achieve listening and reading comprehension. The current study examined the efficacy of self-reference encoding for L2 vocabulary learning. In Experiment 1, native Hebrew speakers learned rare English words with a self-reference task and a control condition of translation repetition. In Experiment 2, participants learned with the same self-reference task and a control task of semantic processing. Across both experiments, memory was higher in the self-reference condition in both an immediate and a delayed test one week later. Thus, self-reference might be adopted as a learning tool in L2 vocabulary learning. Further, we demonstrate the contribution of self-reference to learning new information, going beyond previous demonstration of its positive impact on episodic encoding of known information.

## Introduction

Learning a foreign language is a complex process that requires mastering various linguistic skills. One of the major challenges is building a large vocabulary, since learners must acquire thousands of words. Specifically, in order to understand daily spoken discourse, learners need to be familiar with the 2,000–3,000 most frequent word families (van Zeeland & Schmitt, 2013), and to comprehend texts in a variety of subjects, an even larger vocabulary of the 8,000–9,000 most frequent word families is necessary (Nation, 2006).

In light of the central role of L2 vocabulary knowledge, various methods have been proposed to improve vocabulary learning. Among them are spaced repetitions (Nakata, 2015), retrieval practice (Rice & Tokowicz, 2020), the word parts technique (Wei & Nation, 2013), the keyword technique (Pressley, 1977), and more. While these methods are effective, exploring new learning methods can be beneficial given the scope of L2 vocabulary needed. Thus, the current study aims to investigate a novel technique for L2 vocabulary learning based on the self-reference effect.

### The self-reference effect

It is easier to remember information encoded in relation to the self than information encoded through other modes, a phenomenon known as the self-reference effect (Bentley et al., 2017; Gilliam & Gutchess, 2024; Glisky & Marquine, 2009; Klein & Loftus, 1988; Leshikar et al., 2015; Rogers et al., 1977; for a meta-analysis, see Symons & Johnson, 1997). Self-reference also enhances learning in educational settings where students learn school materials (for a meta-analysis, see Liu et al., 2024).

Early studies proposed two cognitive mechanisms that underlie the self-reference effect: elaboration and organization (Klein, 2012; Symons & Johnson, 1997). Elaboration involves linking new information to a network of prior knowledge. When learners encode a word by referencing it to the self, it becomes connected to a rich network of self-knowledge, which facilitates memory (Bradshaw & Anderson, 1982; Kendzierski, 1980). Organization involves creating relationships between items in a word list. Referencing a list to the self creates a shared link between the items, enhancing retention (Klein & Kihlstrom, 1986; Mandler, 1967).

Continuing research has shown that self-reference improves memory even without an explicit connection to self-knowledge. Labeling items as owned by learners enhances memory, indicating that mere ownership increases retention of the items (Clarkson et al., 2024; Cunningham et al., 2008). Participants also better remembered the location of words that appeared near their name compared to those near a celebrity's name (Turk et al., 2008). Additionally, an arbitrary association between the self and geometric shapes improves the recognition accuracy of the shapes (Sui et al., 2012), and self-reference also enhances source memory, an aspect of memory acquired implicitly (Rosa et al., 2024; Sweatman et al., 2022). Finally, neuro-imaging studies have shown that different brain networks are involved in self-reference and semantic processing (for detailed descriptions, see Binder et al., 2009; Hu et al., 2016). These studies suggest that self-reference is a potent process which creates a memory bias, probably involving additional mechanisms beyond elaboration and organization.

Research on the self-reference effect described above has demonstrated its efficacy in supporting learners’ ability to episodically encode and remember words they are already familiar with in their L1. Here we extend this line of research to ask whether self-reference can also support acquiring previously unknown words in an L2. Learning a new L2 word is a complex task involving learning the form, the meaning, and the form-meaning connection (Baxter et al., 2021). Therefore, it is unclear whether self-reference is as effective in supporting this type of learning, which differs from the mostly episodic learning examined in previous research. One study examined the efficiency of self-reference for L2 vocabulary learning and found no advantage for self-reference over imagery (Park, 2018). Importantly, in that study participants recalled an experience involving other people manifesting the given adjective words, which may not have appropriately engaged self-reference. The current study examines the efficacy of a direct reference to self-knowledge.

### How to induce self-reference?

Studies have operationalized self-reference in different ways. One classic self-reference task is adjective judgment, in which participants evaluate whether a trait adjective characterizes them ("Does *extrovert* describe you?"; e.g., Glisky & Marquine, 2009; Rogers et al., 1977). Other studies have shown participants pictures of objects (nouns) and asked, “Do you like it?” (Rosa et al., 2024; Sweatman et al., 2022). Another self-reference task is autobiographical elaboration, in which participants link a word to an episodic memory, which is a specific past event related to the word (Klein et al., 1989). Such autobiographical elaboration has been implemented using nouns (e.g., Bellezza, 1984, study 2; Klein & Kihlstrom, 1986) or adjectives (e.g. Bellezza, 1984, study 1; Klein et al., 1989; McDonough & Gallo, 2008).

Since the current study addresses L2 vocabulary learning, the task should be appropriate for applied settings. Specifically, we were interested in implementing a task that can apply to words from different parts of speech (nouns, verbs, adjectives). We were also concerned that autobiographical elaboration may be limited for L2 vocabulary learning since the learner might not have relevant episodic memories for all target words. Therefore, in the current study, we used a sentence-generation task in which participants created a sentence that included the target word. Importantly, participants generated sentences in L2 (English) and did so orally to avoid the complexity of English spelling (Share, 2008).

We defined the self-reference task broadly to be applicable to real-life learning while constraining the options to three distinct categories: personal facts, autobiographical facts, and episodic memory. The personal facts category includes references to general facts about the self in the present ("I *exercise* three times a week"; “I am an *extrovert*”). This category may include adjective judgment when the target word is an adjective, and also may include any other reference to personal facts related to the word. The autobiographical facts category includes general facts about the self in the past ("During high school, I *played* in a chess club"). The episodic memory category includes references to specific past events ("I bought a *wallet* in the market last week").

Thus, in the current study, we use a broad operationalization of self-reference, which we believe has the potential to be more applicable to practical L2 vocabulary learning. However, it does not include references to personal opinions, future plans, or imagery scenarios, which may be additional types of self-reference.

### What is self-reference compared against?

When assessing whether self-reference is an effective encoding method, a key question is what type of control condition it is compared against. Previous studies on L1 memory encoding primarily compared self-reference with semantic processing or reference to another person at varying levels of intimacy (Symons & Johnson, 1997). Here, we explore the effectiveness of self-reference with other tasks widely used for L2 vocabulary learning. In Experiment 1, we compared self-reference to translation production (saying the L1 translation aloud), since learning L2 vocabulary from word lists is highly effective (for a meta-analysis, see Webb et al., 2020).

In Experiment 2, we compared self-reference to semantic processing, which was used as the primary control condition in many L1 self-reference studies (Symons & Johnson, 1997). In semantic processing tasks, the learner expands and engages with the word's meaning, which yields higher memory rates in L1 memory encoding (Craik & Lockhart, 1972; Morris et al., 1977). Notably, the efficacy of semantic processing for L2 vocabulary learning has been debated, and some previous research has demonstrated that semantic processing did not improve the memory of L2 vocabulary (Barcroft 2002, 2003, 2004, 2009; Kida, 2020; Wong & Pyun, 2012). However, the methodology in some of these previous studies has been criticized for the tasks and control conditions used (Lindstromberg, 2020; Rice & Tokowicz, 2020). Thus, it is as yet unclear whether semantic processing is effective for L2 vocabulary learning.

In Experiment 2, we compared self-reference with semantic processing mainly to replicate the basic control condition used in many L1 self-reference studies. Considering the critique of previous research, in the current study participants created sentences that defined the meaning of the word and included the target word within them ("A *banister* is the part on the side of the stairs that you can hold onto for support when going up and down"). This task is similar to the self-reference task in requiring L2 sentence generation and oral production. In summary, the present study examines whether self-reference improves L2 vocabulary learning. We defined self-reference broadly, and compared it to translation production (Experiment 1), and semantic processing (Experiment 2). All materials and data are available on OSF https://osf.io/xepq7/?view_only=cd737049a6204c229f3972e314c38339

## Experiment 1

### Method

#### Participants

Participants were native Hebrew-speaking young adults who reported that they could hold a basic conversation in English. English instruction in Israel is mandatory, begins in third grade, and continues throughout high-school, leading to intermediate levels of proficiency in the language. Participants who declared ahead of time that their English level is close to native were excluded. Additionally, five participants were excluded after participating because they stated in the post-experiment questionnaire that they knew at least four of the 20 words learned during the experiment (see details below). The final sample included 42 participants (33 women, mean age 25 years), who received payment or academic credit (see Table 1).

**Table 1:** Participant characteristics

	English vocabulary size	Working memory capacity
Experiment 1	4,000 (1,910)	4.24 (0.87)
Experiment 2	3,875 (2,512)	4.25 (0.95)

*Note:* There were no significant differences on either measure, both *p* > 0.8

#### Background measures

Given that we conducted two experiments with two separate groups, we measured vocabulary size and working memory to ensure that there were no significant differences between the groups in these background variables, which are known to predict L2 vocabulary learning (Linck et al., 2014; Webb & Chang, 2015).

*Vocabulary test*: Participants completed the Vocabulary Size Test in English (Nation & Beglar, 2007). This multiple-choice test presents a word, and the participant chooses the word closest to its meaning from four options. The test presents ten words from each thousand-word frequency band sequentially and is terminated when the participant responds incorrectly to four words from a given band. The score is the total number of correct answers, which estimates the participant's total vocabulary size by multiplying the score by 100. For instance, a score of 35 suggests the participant knows the most frequent 3,500 words in English.

*Working memory:* Participants competed the Color Span Backwards test (Hasselhorn et al., 2012). This test presents a sequence of colored circles appearing one after another for one second each. Participants are instructed to tap the presented colors in reverse order using keys on the keyboard marked with the different colors. The test begins with a sequence of two colors, and as the test progresses the sequence length increases to a maximum of eight colors. There are three trials for each sequence length. The test is terminated when the participant responds incorrectly to two trials of a given sequence length. The score is the longest sequence length that the participant correctly recalled.

#### Materials

We compiled a list of rare English words, to retain qualities often absent in pseudowords such as form-meaning regularities (Blasi et al., 2016; Reilly & Kean, 2007), and conducted a pilot study to ensure the stimuli were unknown to the intended participants. The study included 39 rare words ranked above 10,000 in the descending frequency rating of the COCA corpus (Davies, 2010), five high-frequency words, and five pseudowords. High-frequency words were included to maintain participants’ alertness and discourage automatic responses. Pseudowords were included to identify participants who overestimated their vocabulary knowledge (see Paulhus, 2002; Paulhus et al., 2003). Participants had the following response options for each word: (1) I know the translation of the word, (2) I know the word but not its translation, or (3) I do not know the word. The questionnaire was distributed online and completed voluntarily. Out of 40 respondents, nine were disqualified for verifying two or more pseudowords as real words. Following the pilot, we excluded two rare words recognized by at least five participants.

From the remaining 37 rare words, we compiled two lists of ten words each, with each list including four nouns, three verbs, and three adjectives. The lists were matched on rank order frequency (based on COCA, *t*(18) = 0.96, *p* > 0.05), on frequency per million (based on SUBTLEX, *t*(18) = 0.34, *p* > 0.05), length in phonemes (*t*(18) = 1.12, *p* > 0.05), and length in letters (*t*(18) = 1.77, *p* > 0.05; see Appendix 1 and Online Supplementary Material are available on OSF https://osf.io/xepq7/?view_only=cd737049a6204c229f3972e314c38339 for the full list of stimuli).

#### Design and experimental tasks

The experiment used a within-participant design, where each participant completed two training conditions, self-reference and translation production, each including ten words. Each training block was preceded by a practice stage containing three high-frequency words – a noun, a verb, and an adjective. The assignment of lists to conditions and the order of blocks were counterbalanced across participants. The order of the target words in each list was randomized for each participant. Participants were not informed about the upcoming memory test.

*The self-reference task:* Participants were instructed to create a sentence in English including the target word, describing a fact or a memory about themselves, and specifically one of three types of sentences: (1) a sentence describing a general fact about themselves in the present (e.g., "I *listen* to music every day on my way to work"), (2) a sentence describing a general fact about their past (e.g., "When I was a teenager, I *listened* to music a lot"), (3) a sentence describing a specific past event (e.g., "Yesterday on my way to work, I *listened* to Leonard Cohen").

Before creating the sentence, participants were exposed to the new target word. In each trial, an English word appeared with its Hebrew translation, in writing (e.g., assiduous – חרוץ), and remained on the screen until participants recorded their responses. Second, participants clicked on a "listen" icon and listened to the English word's pronunciation recorded by a native English-speaking female adult, ensuring exposure to the L2 spoken form. This exposure to the spoken form is essential since for many English words, it is difficult to infer from the written form how to pronounce the word (Share, 2008). Participants listened to the word once and could not listen again. Third, participants clicked on a "speak" icon and vocally produced the English word into a microphone. This stage was intended to prepare participants to use the English word orally in the upcoming sentence-generation task. Fourth, they re-clicked the "listen" icon and listened again to the same recording of the English word they had listened to previously. Finally, participants generated the self-reference sentence in English, using a button to start and stop recording. When pressing the “start recording” button, the English word and its Hebrew translation disappeared from the screen, and only the "stop recording" button remained. When pressing the "stop recording" button the trial ended and the next target word appeared.

Many studies of L2 vocabulary learning include repetitions to facilitate learning (see Rice & Tokowicz, 2020, for a review). Here we decided to present participants with each target word only once, in order to match the designs of research on the self-reference effect conducted in L1 (e.g., Bellezza, 1984; Bentley et al., 2017; Gilliam & Gutchess, 2024; Glisky & Marquine, 2009; Klein & Loftus, 1988; Leshikar et al., 2015; Rogers et al., 1977), to which we wish to compare the current findings. Thus, we avoided multiple repetitions while using a procedure that exposed participants to the written and spoken forms of the words.

*The translation production task*: The procedure was identical to the self-reference condition except in the fifth step. Instead of creating a sentence, participants verbally produced the Hebrew translation of the word.

*Testing*: After completing the two conditions, participants solved simple arithmetic drills for 3 min to create a time gap between training and testing. Then they were presented with an unexpected L2 to L1 translation test, as most L1 studies on the self-reference effect implemented a surprise test (Symons & Johnson, 1997). We used the L2-to-L1 test because it is easier than L1-to-L2 retrieval (González-Fernández & Schmitt, 2020), as participants only needed to recognize the English words rather than retrieve them. Given that participants were exposed to the words only once, we opted for the easier test. In each test trial, one of the 20 English words from training appeared on the screen with a question mark (e.g., *assiduous* - ?), then participants clicked an icon and listened to the English word, to match the presentation condition in the learning stage. Then, participants had to say the Hebrew translation into a recording microphone using a button to start and finish the recording. We opted for spoken responses to avoid potential spelling or typing errors that could alter the meaning of words. When participants could not recall the translation, they clicked a "Skip the word" button.

*Post-experiment questionnaire:* To ensure that participants were not previously familiar with the words presented in the study, they completed a post-experiment questionnaire that included the 20 target words, marking an "X" next to each word they knew before the experiment. Based on this self-report, we excluded from the analyses words known by the participants prior to the experiment. We also disqualified participants who were previously familiar with four or more words.

#### Procedure

In the first session, participants completed the experimental task, and the immediate memory test. The second session occurred 7 days later, and included a second L2 to L1 translation task, the working memory and the vocabulary tasks.

### Results

#### Data cleaning and coding

Participants created a total of 420 self-reference sentences. Of these, 16 sentences were excluded from the analyses as participants indicated in the post-experiment questionnaire that they were familiar with the target words. From the remaining 404 sentences, 338 met the self-reference criteria as defined above (see Appendix 2 for details). An additional 37 sentences were references to family members, friends, or pets. We include these sentences in the analysis since references to close relatives are similar or the same as self-reference (Hamami et al., 2011; Rosa et al., 2024; Serbun et al., 2011; Symons & Johnson, 1997). Another 29 sentences did not meet the self-reference condition because they either lacked self-referential content or did not include the target words. Despite this, we included these sentences in the memory analyses since they were created under the self-reference condition, which presumably kept the concept of self in mind when the sentences were formulated. In the translation production condition, nine items were excluded from the analyses, because participants marked them as familiar in the post-experiment questionnaire.

Performance on the memory test was strictly coded. Namely, answers were marked as correct only if they exactly matched the word provided during the learning phase (alternative inflections and derivations were marked as errors).^1^

#### Memory rates

**Fig. 1:**
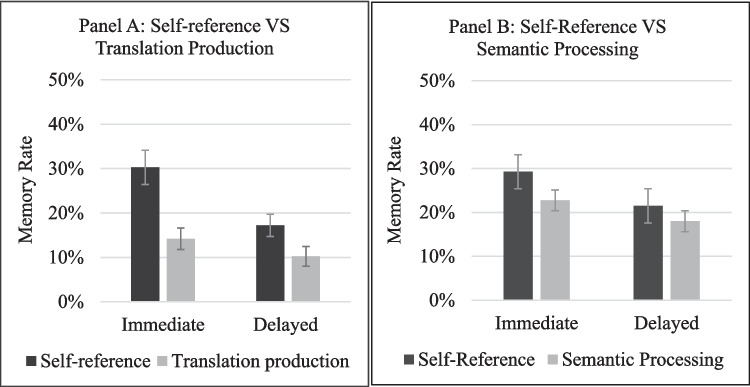
Memory rate by learning condition, in Experiment 1 (**Panel 1**), and Experiment 2 (**Panel B**). Error bars represent the standard error of the mean (SEM)

Memory scores were calculated as percentages, due to the elimination of previously familiar words for some participants (see Table 1 for averages and SDs, and Fig. 1, Panel A). Memory rates were analyzed using a two-way repeated-measures ANOVA, with condition and testing point as within-participant variables. Memory rate was significantly higher in the self-reference condition than in the translation condition, *F*(1,41) = 20.99, *MSE* = 0.026, *p* < 0.001, *η*^*2*^_*p*_ = 0.34, and was significantly higher in the immediate test compared to the delayed test, *F*(1,41) = 38.14, *MSE* = 0.008, *p*<0.001, *η*^*2*^_*p*_ = 0.48. These main effects were modified by a significant two-way interaction *F*(1,41) = 11.77, *MSE* = 0.007, *p* < 0.01, *η*^*2*^_*p*_ = 0.22. Importantly, follow-up paired-samples t-tests demonstrated that memory was significantly higher in the self-reference than in the translation condition both at the immediate (*t*(41) = 4.63, *p* < 0.001, *d* = 0.71) and at the delayed memory tests, (*t*(41) = 3.38, *p* < 0.01, *d* = 0.52).

### Discussion

In Experiment 1, memory for new words was better following self-reference encoding than following translation production in both the immediate and delayed tests. This finding suggests that processing a novel item in reference to the self might enhance its encoding, thus leading to improved memory of the word. However, in the translation production condition, participants did not produce a context sentence including the target word as they did in the self-reference condition. Therefore, the observed difference might be due to the richness of encoding rather than self-reference alone. To further explore this issue, we conducted Experiment 2, in which the control condition was semantic processing, which has been used previously as a control condition in L1 self-reference studies. The semantic processing task matched the self-reference task in terms of mental processing requirements and L2 output generation, differing only in the aspect of self-referential processing.

## Experiment 2

### Method

#### Participants

Participants were 48 individuals sampled from the same population and according to the same criteria as in Experiment 1. Eight participants were excluded based on the post-experiment questionnaire. The final sample included 40 participants (31 women, mean age 24 years), who received course credit or payment for their participation (see Table 1 for participant characteristics).

#### Materials, procedure, data coding, and cleaning

Experiment 2 used the same materials, procedures, and data cleaning and coding approaches as Experiment 1. The main difference was in the learning phase, which included a semantic processing task instead of the translation task. The semantic processing condition was similar to the self-reference condition, except in the fifth stage, where participants were instructed to create a sentence in English that defines the meaning of the word and to include the word in the sentence.^2^

### Results

In this experiment, participants created 400 self-reference and 400 semantic processing sentences. Following the post-experimental questionnaire, 18 sentences were excluded from the analyses in each condition (self-reference and semantic processing) since participants reported being familiar with the target words before the experiment. In the semantic processing condition, 12 sentences did not include the target word in the sentence or included self-referential content. Nonetheless, these were included in the analysis following the same criteria as in Experiment 1 (see Appendix 2).

Memory rates were calculated as percentages as in Experiment 1 (see Table 2 and Fig. 1, Panel B). Data were analyzed using a two-way repeated-measures ANOVA, with training condition and testing point as within-participant variables. Overall, memory rates in the self-reference condition were significantly higher compared to the semantic processing condition, *F*(1,39) = 5.05, *MSE* = 0.02, *p <* 0.05,*η*^*2*^_*p*_ = 0.11, and memory at the immediate test was significantly higher than in the delayed test *F*(1,39) = 21.84, *MSE* = 0.007, *p* < 0.001, *η*^*2*^_*p*_ = 0.36. The two-way interaction was not significant *F*(1,39) = 1.5, *MSE* = 0.006, *p* = 0.227, *η*^*2*^_*p*_ = 0.03.

**Table 2:** Means (SD) of memory rate by condition for the two experiments

Experiment 1		
	Self-reference	Translation production
Immediate memory test	30.3% (25.2%)	14.3% (15.8%)
Delayed memory test	17.0% (16.3%)	10.2% (14.3%)
Experiment 2		
	Self-reference	Semantic processing
Immediate memory test	29.3% (21.2%)	22.7% (22.1%)
Delayed memory test	21.5% (19.6%)	18.0% (20.7%)

### Discussion

Experiment 2 compared self-reference to semantic processing, with both tasks requiring participants to create sentences in L2. Consistent with Experiment 1, the results demonstrated an advantage for self-reference, further supporting its effectiveness in L2 vocabulary learning. These findings align with the broader literature that highlights the effectiveness of self-reference in various cognitive and educational domains.

## General discussion

The current study examined the effectiveness of self-reference for L2 vocabulary learning. Participants learned rare English words under self-reference and a control condition. Across both control conditions examined, namely translation production and semantic encoding, self-reference encoding resulted in better memory. This advantage was evident immediately after learning, and remained significant 1 week later.

Whereas previous research demonstrated the benefits of self-reference in episodic memory tasks in the L1 (Liu et al., 2024; Symons & Johnson, 1997), here we extend these findings and demonstrate the efficacy of self-reference to learning novel vocabulary in the L2. The task of learning new words requires participants to learn the form, the meaning, and the form-meaning connection (Baxter et al., 2021), and not only the episodic presence of a previously known item in the experimental setting. Given this complexity, it is not obvious that self-reference processing, which might require additional cognitive resources, would be effective. Indeed, there are claims that any form of semantic processing does not promote the learning of new L2 vocabulary (Barcroft, 2002, 2003, 2004, 2009). Notably, the current results demonstrate the efficacy of self-reference for L2 vocabulary learning, in comparison to both translation production and semantic processing. These results can be explained by the theoretical accounts mentioned above, suggesting that the cognitive system prioritizes self-relevant information (Cunningham et al., 2008; Sui et al., 2012; Turk et al., 2008), and that self-reference promotes elaboration and organization, which improve memory (Klein, 2012; Symons & Johnson, 1997).

The findings of the current study, namely a positive effect of self-reference on L2 vocabulary learning, open up many avenues for future research. First, here we used an oral vocabulary learning task, but it is important to explore whether the effect is maintained in writing tasks as well. Speaking in a foreign language can induce anxiety, as speakers may fear their speech will be judged negatively for inaccuracies, and such anxiety can interfere with learning (Mak, 2011; Miskam & Saidalvi, 2019; Teimouri et al., 2019). In addition, in many foreign language instructional settings, students engage in writing more than speaking. Thus, future studies could explore whether self-reference is an effective tool for L2 learning in the written modality as well.

Second, in the current study, the implemented task was limited to only a few types of self-reference, specifically personal facts about the present or the past and episodic memories. Future studies could explore the effectiveness of other self-reference types, such as references to personal opinions or imaginary scenarios involving the self. Along similar lines, another method worth investigating is the use of personal pronouns like "I," "you," or "your" in texts and learning assignments, as they have been shown to enhance certain aspects of learning. For example, there are improvements in learning outcomes when math exercises included the word "you" (Cunningham et al., 2024), in spelling learning when students wrote sentences starting with "I" (Turk et al., 2015), and in anatomy learning when texts include the word "your" ("your eye" instead of "the eye"; Dutke et al., 2016). Future research could further explore whether learning tasks incorporating personal pronouns improve vocabulary learning.

Finally, future studies could examine whether stimuli, learner and task characteristics might modulate the effect of self-reference. For example, word valence influences the self-reference effect in L1 (see Argembeau et al., 2005; Durbin et al., 2017), and a similar effect may extend to L2 word learning. One limitation of the current study is that word valence was not controlled. Specifically, we were unable to match the valence of the rare words used here, since most of them do not appear in existing valence databases. For instance, the NRC-VAD database (Mohammad, 2018), which includes 20,000 words, contains valence ratings for only nine of the 20 words used in this study, while the Warriner et al. (2013) database provides data for only eight of the 20 words. Future research could investigate whether valence modulates the self-reference effect in L2 word learning.

Another avenue for research involves exploring individual differences in the self-reference effect for L2 learning. Factors such as working memory, vocabulary size, and emotional traits like anxiety may modulate the effectiveness of self-referential learning in L2 (see Moses-Payne et al., 2022, on social anxiety and the self-reference effect). Finally, future research could also examine how repetitions in learning, which are commonly implemented in L2 vocabulary instruction (e.g. Rice & Tokowicz, 2020), might modulate the effect of self-reference, testing the boundary conditions for its efficacy.

## Conclusion

The current study showed that self-reference improves vocabulary learning in a foreign language. These findings may be useful for foreign language learning since achieving proficiency requires learning thousands of new words (Nation, 2006). Self-reference can serve as a practical learning tool to achieve this goal. In addition, the current findings extend our understanding of self-reference as a learning mechanism, by showing that it supports the acquisition of new knowledge, and not only episodic encoding of existing knowledge.

## Data Availability

All experimental stimuli and data of the current study are available via the Open Science Framework (OSF) repository at: https://osf.io/xepq7/?view_only=cd737049a6204c229f3972e314c38339 Neither of the experiments was pre-registered.

## Appendix 1: Stimuli

**Table:**

List 1						
Letters	Phonemes		SUBTLEX Log frequency per million	COCA frequency rank	Part of speech	Word
8	8		1.31	22992	Noun	banister
6	6		0.31	45680	Noun	flagon
8	6		0.27	37797	Noun	squeegee
5	4		0.61	19381	Noun	swoon
7	6		0.02	43631	Verb	flummox
6	5		-	44128	Verb	garble
7	6		0.08	26120	Verb	mollify
7	5		0.98	16680	Adjective	callous
9	9		0.04	36736	Adjective	corpulent
8	7		0.04	40017	Adjective	obdurate
7.1 (1.2)	6.2 (1.5)		0.4 (0.46)	33316 (10965)		Mean (SD)
List 2						
Letters	Phonemes		SUBTLEX Log frequency per million	COCA frequency rank	Part of Speech	Word
9	9		0.49	14198	Noun	conundrum
10	9		0.57	23756	Noun	contrition
5	4		0.75	21410	Noun	ladle
7	5		1.47	18391	Noun	satchel
8	7		0.04	26417	Verb	disburse
7	6		0.16	36124	Verb	forbear
9	7		0.02	27066	Verb	vacillate
9	7		0.04	37386	adjective	assiduous
10	9		0.82	38483	adjective	nincompoop
8	7		-	44821	adjective	timorous
8.2 (1.5)	7 (1.7)		0.48 (0.48)	28805 (9933)		Mean (SD)

## Appendix 2: Numbers and percentage of sentence types created by experiment

**Table:**

**Self-reference task**			
		Experiment 1	Experiment 2
Total approved sentences		404	382
Personal facts		50.5% (204)	51.3% (196)
Autobiographical facts		7.9% (32)	13.9% (53)
Episodic memories		25.3% (102)	24.6% (94)
Close relatives		9.2% (37)	7.1% (27)
Not follow instructions		7.2% (29)	3.1% (12)
**Semantic processing task**			
			Experiment 2
Total approved sentences			382
Semantic processing			96.9% (370)
Not follow instructions			3.1% (12)

## Footnotes

1 A single exception was made for the word “corpulent.” The Hebrew translation presented to participants was “Adam Shamen,” meaning a fat person, but we also accepted responses where the word “Adam” (person) was replaced with its Hebrew synonyms “Ben Adam” or “Ish.”

2 Following each sentence, in both the semantic and the self-reference conditions, participants rated the difficulty of creating the sentence on a scale of 1–10. There was no difference in these ratings between conditions (p=0.8), alleviating concerns of difficulty or effort as confounding variables leading to the self-reference effect.
