# Correction: Self-reference promotes vocabulary learning in a foreign language

**DOI:** 10.3758/s13423-025-02762-x

**Published:** 2025-12-08

**Authors:** Shimon Pruss, Avi Karni, Anat Prior

**Affiliations:** 1 Department of Learning Disabilities, Faculty of Education, University of Haifa, 199 Abba Hushi Blvd, Haifa, Israel; 2 Edmond J. Safra Brain Research Center for the Study of Learning Disabilities, University of Haifa, Haifa, Israel

**Correction: Psychonomic Bulletin & Review (2025) 32:2104-2113**

https://doi.org/10.3758/s13423-025-02674-w

The appendix of this paper was presented incorrectly. The corrected version appears here.

**Appendix 1:** Stimuli

**Table:** 

List 1					
Word	Part of speech	COCA frequency rank	SUBTLEX Log frequency per million	Phonemes	Letters
banister	Noun	22992	1.31	8	8
flagon	Noun	45680	0.31	6	6
squeegee	Noun	37797	0.27	6	8
swoon	Noun	19381	0.61	4	5
flummox	Verb	43631	0.02	6	7
garble	Verb	44128	-	5	6
mollify	Verb	26120	0.08	6	7
callous	Adjective	16680	0.98	5	7
corpulent	Adjective	36736	0.04	9	9
obdurate	Adjective	40017	0.04	7	8
Mean (SD)		33316 (10965)	0.4 (0.46)	6.2 (1.5)	7.1 (1.2)
List 2					
Word	Part of Speech	COCA frequency rank	SUBTLEX Log frequency per million	Phonemes	Letters
conundrum	Noun	14198	0.49	9	9
contrition	Noun	23756	0.57	9	10
ladle	Noun	21410	0.75	4	5
satchel	Noun	18391	1.47	5	7
disburse	Verb	26417	0.04	7	8
forbear	Verb	36124	0.16	6	7
vacillate	Verb	27066	0.02	7	9
assiduous	adjective	37386	0.04	7	9
nincompoop	adjective	38483	0.82	9	10
timorous	adjective	44821	-	7	8
Mean (SD)		28805 (9933)	0.48 (0.48)	7 (1.7)	8.2 (1.5)

**Appendix 2:** Numbers and percentage of sentence types created by experiment

**Table:** 

Self-reference task		
	Experiment 1	Experiment 2
Total approved sentences	404	382
Personal facts	50.5% (204)	51.3% (196)
Autobiographical facts	7.9% (32)	13.9% (53)
Episodic memories	25.3% (102)	24.6% (94)
Close relatives	9.2% (37)	7.1% (27)
Not follow instructions	7.2% (29)	3.1% (12)
Semantic processing task		
		Experiment 2
Total approved sentences		382
Semantic processing		96.9% (370)
Not follow instructions		3.1% (12)

